# Simple Model of Liquid Water Dynamics

**DOI:** 10.1021/acs.jpcb.3c05212

**Published:** 2023-09-06

**Authors:** Tomaz Urbic, Ken A. Dill

**Affiliations:** † Faculty of Chemistry and Chemical Technology, University of Ljubljana, Večna pot 113, SI-1000 Ljubljana, Slovenia; ‡ Laufer Center for Physical and Quantitative Biology, and Departments of Chemistry and of Physics & Astronomy, Stony Brook University, Stony Brook, New York 11794-5252, United States

## Abstract

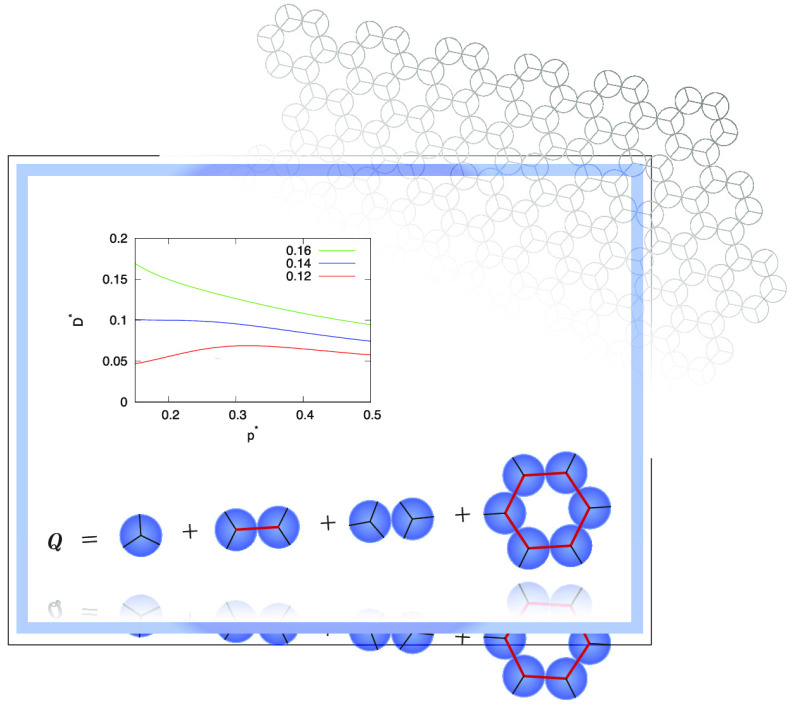

We develop an analytical statistical-mechanical model to study the dynamic properties of liquid water. In this two-dimensional model, neighboring waters can interact through a hydrogen bond, a van der Waals contact, or an ice-like cage structure or have no interaction. We calculate the diffusion coefficient, viscosity, and thermal conductivity versus temperature and pressure. The trends follow those seen in the water experiments. The model explains that in warm water, heating drives faster diffusion but less interaction, so the viscosity and conductivity decrease. Cooling cold water causes poorer energy exchange because water’s ice-like cages are big and immobile and collide infrequently. The main antagonism in water dynamics is not between vdW and H bonds, but it is an interplay between both those pair interactions, multibody cages, and no interaction. The value of this simple model is that it is analytical, so calculations are immediate, and it gives interpretations based on molecular physics.

## Introduction

Liquid water has anomalous aspects of its energetic, volumetric, and dynamic properties relative to simpler liquids like argon.^1^ Water’s properties and anomalies appear to derive from water’s relatively unique molecular structure. Water combines hydrogen bonding with van der Waals interactions with incommensurate tetrahedral and spherical symmetries. This has posed a challenge for statistical-mechanical theories of its liquid properties.^2−14^ We have recently developed such a statistical-mechanical theory for water’s equilibrium properties that treats H bonding and vdW interactions together. Herein, we use that approach to study water’s dynamic properties. Some of the anomalous dynamic properties include the breakdown of the Stokes–Einstein relation^15,16^ and the non-Arrhenius to Arrhenius dynamic crossover at low temperatures.^17−21^

Herein, we adopt a Mercedes-Benz-like model of water, which has previously been studied in 2D and 3D^22−24^ to study the dynamic properties of pure water. The model was introduced in the 1970s by Ben-Naim.^25−28^ The MB models of water are toy models but have the advantage that can explain in a simple way the interplay of thermodynamic properties and angle-dependent potential. The analytical theories for MB-like models allow the inclusion of orientation-dependent hydrogen bonding within a framework that is simple and nearly analytical. According to the 2D MB model, each water molecule is a Lennard–Jones disk with three arms, oriented as in the Mercedes-Benz logo, to mimic the formation of hydrogen bonds. In a statistical-mechanical model, which is based on 2D Urbic and Dill’s (UD) model^22^ being directly descendant from a treatment of Truskett and Dill (TD), who developed a nearly analytical version of the 2D MB model,^29,30^ each water molecule interacts with its neighboring waters through a van der Waals interaction and an orientation-dependent interaction that models hydrogen bonds. Herein, we extended the theory to calculate dynamics properties like diffusivity, viscosity, thermal conductivity, etc. The new version of the theory can be used in all liquid regions of the 2D MB model, including supercooled where computer simulations cannot obtain dynamics properties due to crystallization and convergence problems.

In this article, we start from an analytical 2D UD theory of water.^22^ A partition function for a water molecule in the bulk of different states of the water molecule (hydrogen bonded, cage, van der Waals, and open) is written and static properties of the bulk water are calculated, and the details are provided in the section on Theory. From the bonding of the pair of water molecules we determine diffusivity and from further relations other dynamic properties. In the section on Results and Discussion, we show and discuss the results and summarize everything in the section on Conclusions.

## Theory

### Model for the Equilibrium Water

**Figure 1:**
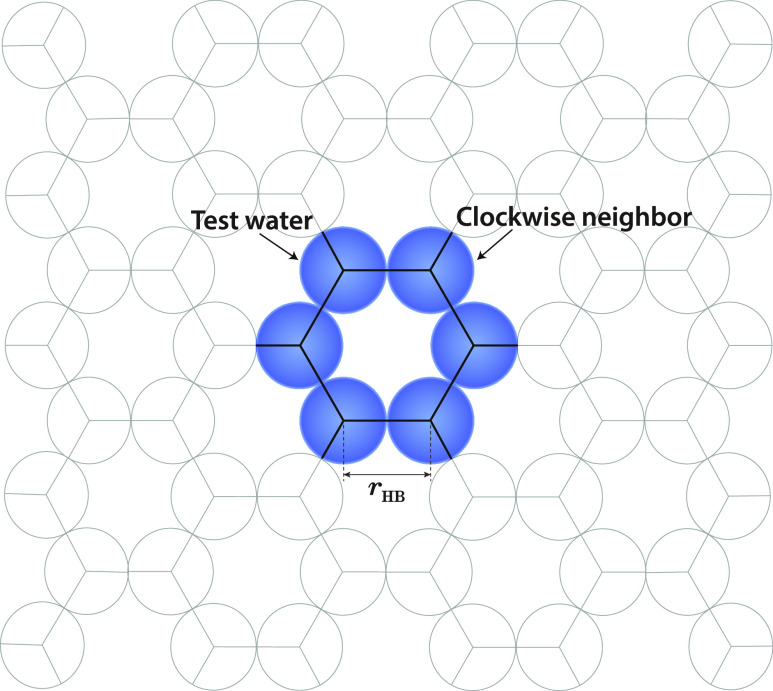
Bookkeeping is done on the underlying lattice to avoid double counting of three-body interactions. The lattice is the hexagon of the ice-like structure. The figure shows a pair interaction used for calculations.

**Figure 2:**
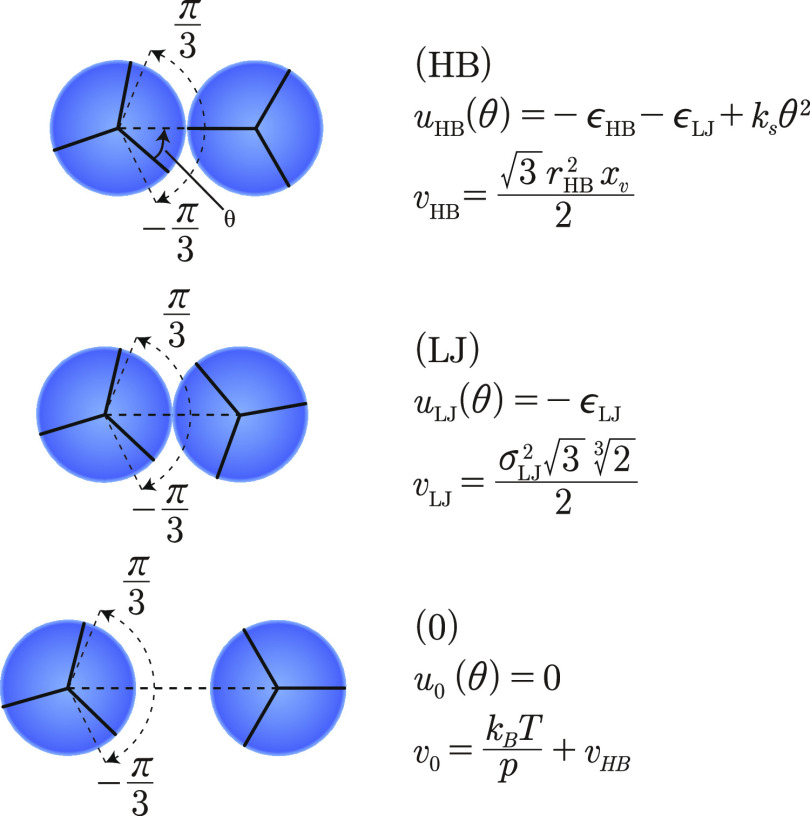
Three model states: hydrogen bonded (HB), Lennard–Jones (LJ) bonded, and nonbonded (0).

Herein, we are briefly reviewing the UD theory.^22^ The system of water consists of *N* molecules. The theory is made for a single water molecule in the hexagon and the relationship of that water to its clockwise neighbor (see Figure 1). The water can form a hydrogen bond (HB), a Lennard–Jones (LJ) contact, or no interaction at all (0). Figure 2 shows these three possible relationships. We compute the isothermal–isobaric statistical weights, Δ_HB_ of the hydrogen-bonded molecules, Δ_LJ_ of the van der Waals contacts, and Δ_o_ of the unbonded population as functions of temperature, pressure, and interaction energies.^22^

#### Hydrogen-Bonded State

**Figure 3:**
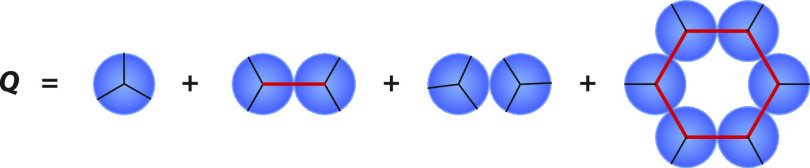
Water partition function for pure water. It has four components: one for noncontacting water molecules, one for H-bonding water pairs, one for LJ pairs, and one for cage-like structures.

Herein, the test water molecule points one of its three hydrogen-bonding arms at an angle θ to within π/3 of the center of its clockwise neighbor water, and it forms a hydrogen bond. The energy of interaction of the test water with its clockwise neighbor depends on orientation and is given by
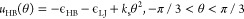
1where ϵ_HB_ is the maximal strength of a hydrogen bond, ϵ_LJ_ is the LJ contact energy, and *k*_s_ is the angular spring constant that changes the strength of HB with orientation. This type of hydrogen bond is a weak hydrogen bond (Figure 3).

#### Lennard–Jones Contact State

Herein, the test water molecule forms only an LJ contact with its clockwise neighbor water. The energy is independent of orientation.
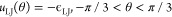
2

#### Noninteracting State

Herein, the test water has no interaction with its clockwise neighbor.
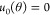
3How to get the total partition function for each hexagon and all of the relative details are explained in previous references^22^ and summarized in the SI.
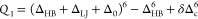
4where δ = exp(−*β ϵ*_c_) is the Boltzmann factor for the cooperativity energy ϵ_*c*_ that applies only when six water molecules all collect together into a full hexagonal cage. Δ_c_ is the Boltzmann factor for a cooperative hexagonal cage. It differs from Δ_HB_ only in that the former uses the hexagonal cage volume per molecule, *v*_c_, while the latter uses the liquid water hydrogen-bonding volume per molecule, *v*_HB_. We combine the Boltzmann factors for the individual water molecules to get the partition function *Q* for the whole system of *N* particles.
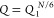
5where the factor *N*/6 accounts for the three possible interaction sites per water molecule and corrects for double counting the hydrogen bonds. The populations of the states *i* = 1 (HB), 2 (LJ), 3(0), and 4(c) can be calculated as
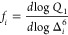
6

From the partition function, all other thermodynamic properties below are obtained as described previously^29,30^ and given in the SI. For all of the model calculations, we used the following parameters: ϵ_HB_ = 1, *r*_HB_ = 1, vdW: ϵ_LJ_ = 0.1, σ_LJ_ = 0.7 (unchanged from Truskett and Dill^29−31^ and the MB model^32^), *k*_s_ = 10, and ϵ_c_ = 0.03.

### Model for the Dynamic Properties

Diffusion processes occur in fluid or gas whenever a property is transported in a manner resembling a random walk. If we assume that the water molecules are doing random walk, we can approximate the diffusion of our molecules in 2D with
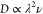
7where λ is the step size and ν is the step frequency. We then compute the diffusion coefficient of water as the weighted average over all of the different individual bond components,
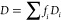
8where *D*_*i*_ = λ_*i*_^2^ν_*i*_ are the diffusion coefficients for HB, c, LJ, and 0 state of water.

The different bond components have different step sizes. For HB and c states, we approximate it as the distance of HB interaction, for LJ state as LJ contact, and for 0 state to average distance between molecules in 0 state.
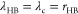
9
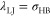
10
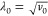
11The step frequency is equal to Boltzmann factor.
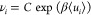
12where *C* is the constant taking care of the units only. The average bonding energies for each state are
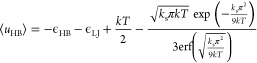
13
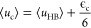
14
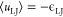
15
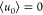
16

From the computed diffusion coefficient *D*, we can readily calculate the viscosity of this model of water as^33^
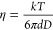
17for temperature *T* and average diameter *d* of water,
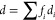
18For water molecules in states HB, LJ, and 0, we used the diameter of the molecule equal to *d*_HB_ = *d*_LJ_ = *d*_0_ = *r*_HB_ while for state s waters form hexagons and the diameter of hexagon state we use equal to *d*_c_ = 2*r*_HB_.

We then also computed the thermal conductivity and thermal diffusivity from this model. For this, we require the speed of sound, which is given by
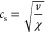
19We obtain the thermal conductivity using a modification of Bridgman’s equation^34^
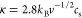
20and thermal diffusivity as
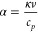
21

## Results and Discussion

In this section, we give theory predictions for how the dynamic properties depend on temperature, pressure, and density. As has been done previously,^22−24^ we present our results below in dimensionless units, normalized to the strength of the optimal HB, ϵ_HB_, and HB separation, *r*_HB_ (T* = *k*_B_*T*/|ϵ_HB_|, u* = *u*/|ϵ_HB_|, V* = *V*/*r*_HB_^2^, and p* = *pr*_HB_^2^/|ϵ_HB_|). Our objective here is to explain qualitatively the trends in experimental data based on the model physics. We cannot compare quantitatively because the model is 2D, while the data is in 3D, meaning that the geometries of the molecules and the units of their properties are different.

**Figure 4:**
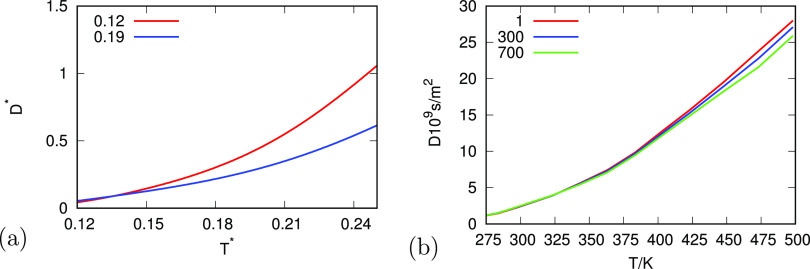
Relationship of the water diffusion coefficient as a function of temperature for different pressures for (a) analytical model and (b) experiment.^35^

Herein, we give the predictions and physical interpretations from the model. Figure 4 compares the predicted dependence of *D* = *D*(*T*) on temperature for liquid water across its liquid range with experiments. Not surprisingly, water’s diffusion gets faster at higher temperatures because more molecules surmount the kinetic barrier to breaking water–water bonding. In cold water, the bond-breaking is mostly of H-bonds; in hotter water, the steeper slope of *D*(*T*) comes from the lower barrier to breaking Lennard-Jones water–water contacts. See different contributions of different populations in the SI.^38^

**Figure 5:**
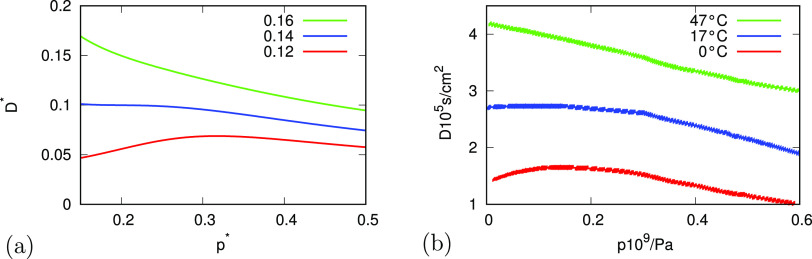
Relationship of the water diffusion coefficient as a function of pressure for different temperatures for (a) analytical model and (b) experiment.^36,37^

Figure 5 compares the pressure dependence, *D* = *D*(*p*), predicted versus experiments, for different temperatures. Higher-temperature water (green curves) is much like a normal Lennard-Jones liquid—the effect of pressure is mainly to squeeze molecules together, reducing their water’s diffusion speed. In contrast, cold water has two pressure regimes. At low pressures, increasing the pressure increases the water’s diffusion rate because it breaks H-bonded cage structures, freeing up waters from those constraints and increasing 0 population. At higher pressures, water’s cages have largely been crunched into a dense LJ liquid as well as nonbonded states and diffusion gets slower with further pressure.

**Figure 6:**
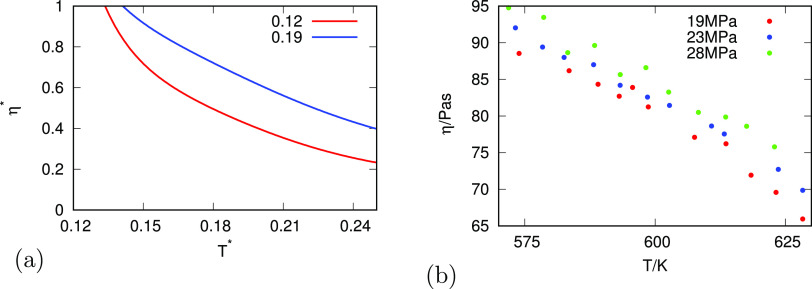
Relationship of the dynamic viscosity of water as a function of temperature for different pressures for (a) analytical model and (b) experiment.^40^

Figure 6 shows the dependence of viscosity η = η(*T*) on temperature, from theory and experiment (up to water’s critical temperature). The physics is the same as that described above for *D*(*T*), to which η(*T*) is inversely related (see eq 17). In the SI, we have also plotted water diffusion as a function of water’s viscosity divided by temperature.

**Figure 7:**
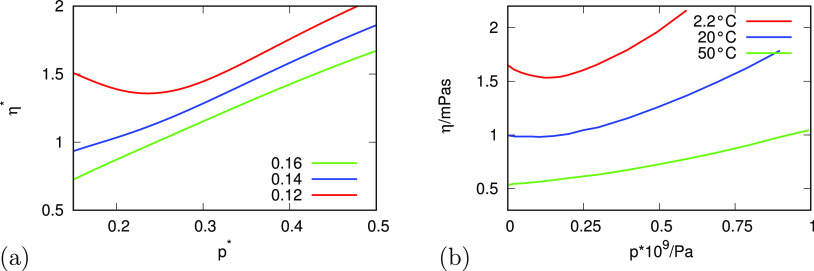
Relationship of the dynamic viscosity of water as a function of pressure for different temperatures for (a) analytical model and (b) experiment.^39,41^

Figure 7 gives the pressure dependence of viscosity η = η(*p*), theory, and experiments. Again, the explanation is the same as for *D*(*T*, *p*), because of the simple inverse relationship, eq 17.

In the SI, we have plotted the water’s diffusivity versus η/*T* plot. This shows the difference in comparison to Stokes’ law. Normal Lennard-Jones fluids have one line because the Stokes’ law is valid in the whole range. For water and its HB and cage states, we no longer have one line, but different regions.

The investigation discerns that the primary contributions to the total diffusion arise from two distinct populations: free particles and LJ particles. By analyzing the temperature and pressure dependencies of these different populations, a more profound understanding of the diffusion process emerges. By analyzing the data, we can still infer which population serves as the primary contributor. Despite the complexity, it is possible to identify the dominant population influencing viscosity through careful examination of the average water particle size, which is intricately linked to all four quantities. Likewise, when thermal conductivity is scrutinized, the main influencing factors are density and isothermal compressibility, both of which are intricately intertwined with all four population parameters.

**Figure 8:**
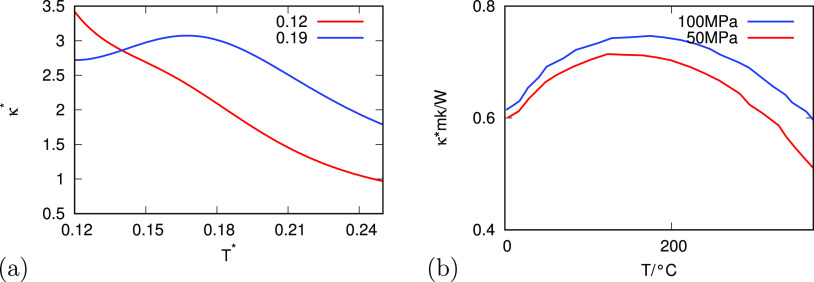
Relationship of the thermal conductivity of water as a function of temperature for different pressures for (a) analytical model and (b) experiment.^42^

Figure 8 shows the thermal conductivity κ = κ(*T*) versus temperature, theory, and experiment (note that experiment data are at pressures higher than the critical pressure of water). κ is the rate at which a material transports heat. The high-temperature decrease of κ is the standard behavior of normal liquids. As the liquid density decreases, it is less effective in transporting heat through collisions. What is more remarkable is water’s decrease in κ with reduced temperature in cold water. The model shows this behavior too for high pressures. We attribute it to the poor ability of water cages, which are relatively large and immobile to collide efficiently to transport heat. At lower pressures, we have only monotonic behavior in our model, but we do not have experimental data to compare. We believe that our model predicts monotonic behavior because there are populations of other states that transport the heat.

We attempted to establish a connection between the roles of cooperatively rearranging regions in anomalous diffusion within the model. However, our efforts were unsuccessful across the range of temperatures and pressures that were explored. Additionally, we did not observe the occurrence of a dynamic crossover.

## Conclusions

In this work, we have developed a theory for the dynamic properties of bulk water within a 2D MB-like model of water. The model assumes three states for each water–water interaction, hydrogen bonded, van der Waals bonded, and nonbonded, and calculations are nearly analytical. The results for diffusivity, viscosity, thermal conductivity, and thermal diffusivity obtained by the analytical theory give the correct general trends as for real water. Theory can easily calculate dynamic properties in the supercooled region of the phase space since we do not have problems with crystallization.

## Supporting Information Available

Detailed description of the theory and additional results for diffusivity and viscosity (PDF)
