# Supplementary material for: Simple Model of Liquid Water Dynamics
Source: J Phys Chem B. 2023 Sep 6;127(37):7996–8001. doi: 10.1021/acs.jpcb.3c05212 (PMC10518820; doi:10.1021/acs.jpcb.3c05212)
Supplement: Supplementary file 1 — jp3c05212_si_001.pdf [file jp3c05212_si_001.pdf]

A Simple Model of Liquid Water Dynamics - Supplementary Information

Tomaz Urbic*,† and Ken A. Dill‡

†*University of Ljubljana, Faculty of Chemistry and Chemical Technology, Večna pot 113, SI-1000 Ljubljana, Slovenia*

‡*Laufer Center for Physical and Quantitative Biology, and Departments of Chemistry and of Physics & Astronomy, Stony Brook University, Stony Brook, NY 11794-5252, USA*

E-mail: tomaz.urbic@fkkt.uni-lj.si

Details of the theory

To compute the isothermal-isobaric partition function, Δ_{HB} we integrate this Boltzmann factor over all the allowable angles and over all the allowable separations (for details, see Urbic and Dill[22]) x and y

$$\Delta_{HB} = c(T) \int\int dxdy \int_{-\pi/3}^{\pi/3} d\theta \exp\left(-(u_{HB} + 2pv_{HB}/3)/k_B T\right). \quad (1)$$

$c(T)$ is the 2D version of the kinetic energy contribution to the partition function, k_B is Boltzmann's constant, T is temperature, p is the pressure, and v_{HB} is volume per molecule. The double integral $\int\int dxdy$ represents the volume over which the second molecule has translational freedom to form a hydrogen bond with the first water and it is equal to effective volume $v_{ef}^{HB} = \sqrt{\pi k_B T}/(2\sqrt{k_s})$. For the perfect hexagon crystal, representing low-pressure ice, the volume of the solid is

$$v_{lds} = \frac{3\sqrt{3}r_{HB}^2}{4}. \quad (2)$$

For high-pressure ice there is another MB water in the center of each hexagonal cage and density here per molecule is,[29]

$$v_{hds} = \frac{\sqrt{3}r_{HB}^2}{2}. \quad (3)$$

The density of liquid water must lie between these limits, we estimate its volume as

$$v_{HB} = \frac{\sqrt{3}r_{HB}^2 x_v}{2}, \quad (4)$$

where $x_v = 1.015$ is simply chosen empirically by fitting the density dependence *vs.* temperature and it is different then reported before.[22] Using these definitions and performing the integration in Equation (1) gives

$$\Delta_{HB} = c(T) v_{ef}^{HB} \exp\left(\frac{\epsilon_{HB} + \epsilon_{LJ} - 2pv_{HB}/3}{k_B T}\right)\sqrt{\frac{k_B T\pi}{k_s}} \mathrm{erf}\left(\sqrt{\frac{k_s \pi^2}{9 k_B T}}\right). \quad (5)$$

The isothermal-isobaric partition function, Δ_{LJ} of this state is given by integrating over angles and positions of the test particle relative to its clockwise neighbor as before,

$$\Delta_{LJ} = c(T) \int\int dxdy \int_{-\pi/3}^{\pi/3} d\theta \exp\left(-(u_{LJ} + 2pv_{LJ}/3)/k_B T\right), \tag{6}$$

where we take the volume occupied by the test water in this state to be

$$v_{LJ} = \frac{\sigma_{LJ}^2 \sqrt{3}\sqrt[3]{2}}{2}. \tag{7}$$

The integral $\int\int dxdy$ represents the translation volume when second molecule forms van der Waals contact with first and is equal to effective volume $v_{ef}^{LJ} = 0.104$. Integrating gives

$$\Delta_{LJ} = \frac{2\pi}{3} c(T) v_{ef}^{LJ} \exp\left(\frac{\epsilon_{LJ} - 2pv_{LJ}/3}{k_B T}\right) \tag{8}$$

The isothermal-isobaric partition function for the non-interacting state is obtained by integrating over translational degrees of freedom,

$$\Delta_0 = c(T) \int\int dxdy \int_{-\pi/3}^{\pi/3} d\theta \exp\left(-2pv_0/3k_B T\right), \tag{9}$$

where v_0 is volume available to the test molecule in this state. Following,[29,30] we compute v_0 using the van der Waals gas approximation,

$$v_0 = \frac{k_B T}{p} + v_{HB}. \tag{10}$$

Integrating over all the ways this state can occur gives

$$\Delta_0 = \frac{2\pi}{3} c(T) \frac{k_B T}{p} \exp\left(\frac{-2pv_0}{3k_B T}\right) \tag{11}$$

Following Truskett and Dill,[29,30] we assume a mean-field attractive energy,,[31] $-Na/v$, among

cages, where a is the van der Waals dispersion parameter (0.03, here) and v is the average molar volume, which we get from Equation (13) below. The true ice cages in water's solid states involve a higher degree of hydrogen-bonding cooperativity than the hydrogen bonding that is just formed pairwise among nearest neighbour waters in the liquid state.

The chemical potential is given by

$$\mu = -\frac{k_B T}{N} \log Q, \tag{12}$$

The molar volume is

$$v = \frac{V}{N} = \left(\frac{\partial \mu}{\partial p}\right)_T = \sum f_i v_i, \tag{13}$$

Results

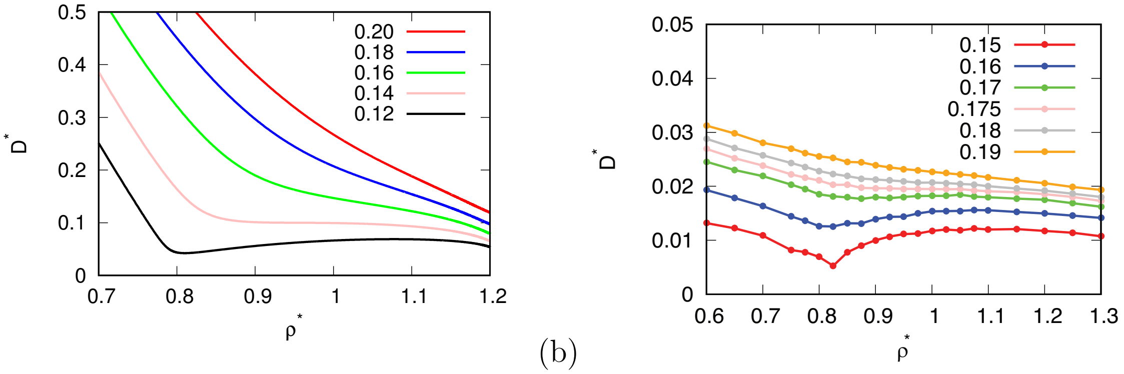

(a) (b)

Figure S1: The relationship of the water diffusion coefficient as a function of (a) density for different temperatures for analytical model and (b) from MC simulations.[36]

The investigation conducted by Urbic et al.[36] reveals that the density dependence exhibits comparable patterns in both the analytical model and pseudodiffusion derived from Monte Carlo simulations (See Figure S1). The calculations also uncovers the existence of an anomalous region. Interestingly, our theoretical predictions indicate that this anomalous region occurs at lower temperatures than those observed in the computer simulations, but

intriguingly, it appears at the same densities in both cases. For a comprehensive visual representation, Figure S2 displays the water diffusion coefficient as a function of inverse temperature. The results show two different regions at low pressures. In Figures S3 and S4, a comprehensive analysis of the temperature and pressure dependencies of different populations' contributions to the overall diffusion process is presented.

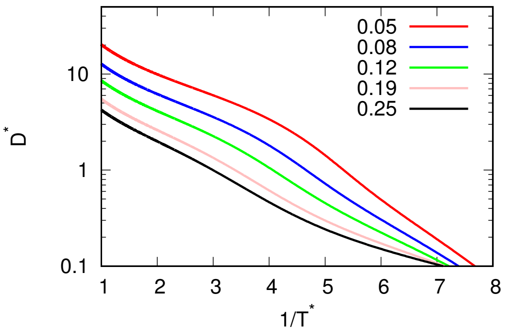

Figure S2: The relationship of the water diffusion coefficient as a function of inverse temperature for different pressures.

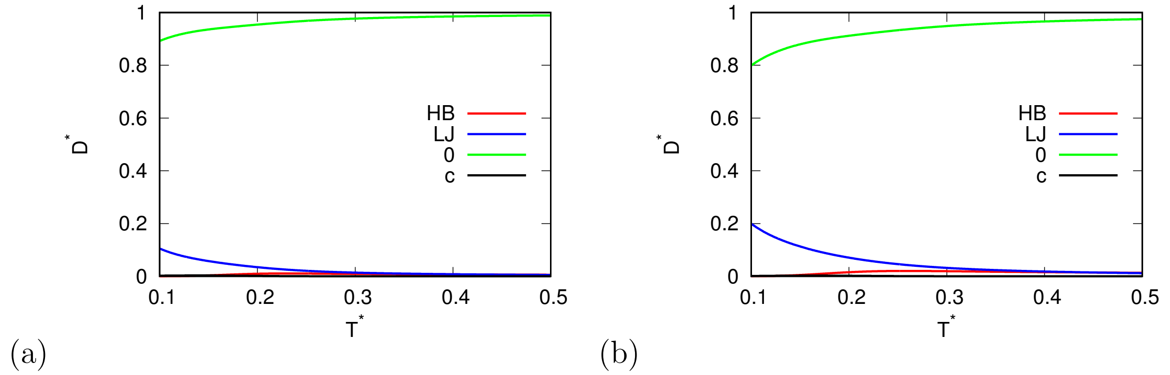

Figure S3: The temperature dependence of the contributions of different populations to total diffusion for (a) $p^* = 0.12$ and (b) $p^* = 0.19$.

References

(1) P. Gallo, K. Amann-Winkel, C. A. Angell, M. A. Anisimov, F. Caupin, C. Chakravarty, E. Lascaris, T. Loerting, A. Z. Panagiotopoulos, J. Russo, J. A. Sellberg, H. E. Stanley,

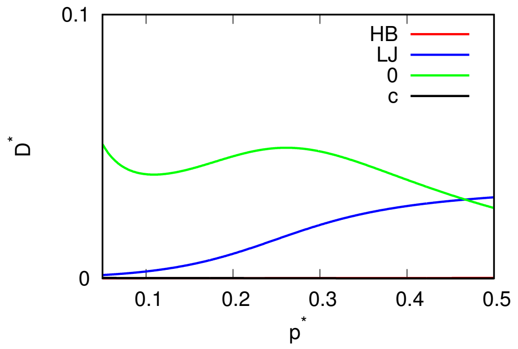

(a)

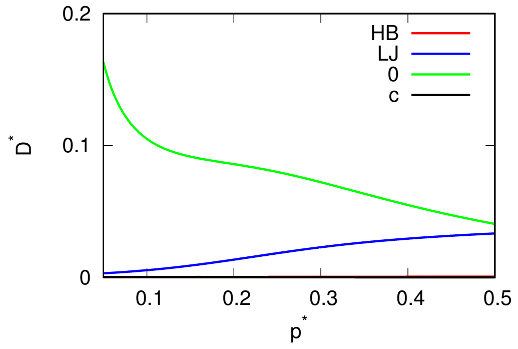

(b)

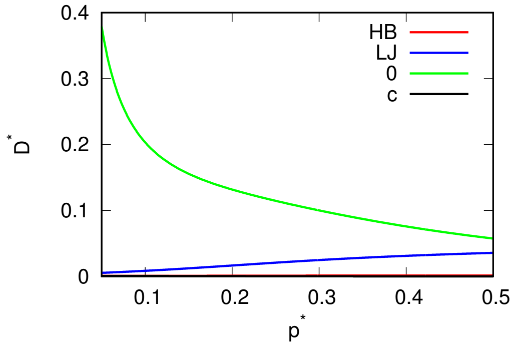

(c)

Figure S4: The pressure dependence of the contributions of different populations to total diffusion for (a) $T^* = 0.12$, (b) $T^* = 0.14$ and (c) $T^* = 0.16$.

H. Tanaka, C. Vega, L. Xu and L. G. M. Pettersson, Chem. Rev. **116**, 7463 (2016), Water: A Tale of Two Liquids.

(2) D. Eisenberg and W. Kauzmann, *The structure and properties of water* (Oxford University Press, Oxford, 1969).

(3) F. Franks., Ed. *Water, a Comprehensive Treatise*, (Plenum Press, New York, 1972–1980) Vol. 1–7.

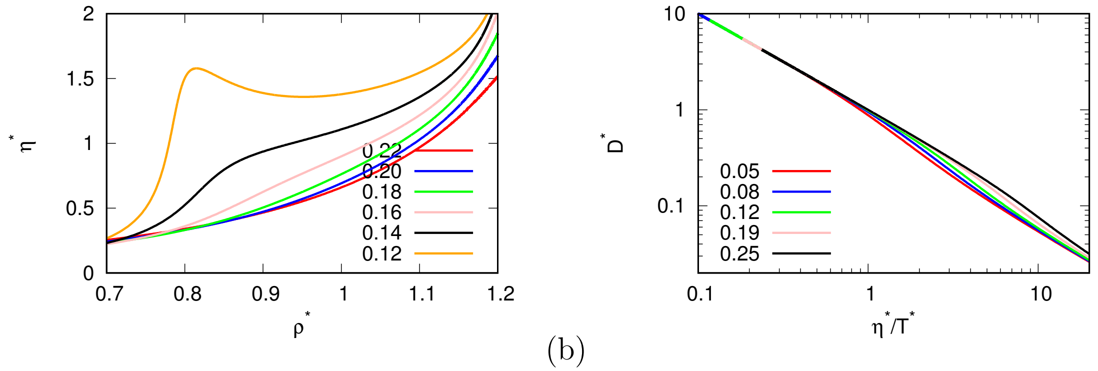

(a) (b)

Figure S5: The relationship of the water viscosity as a function of (a) density for different temperatures for analytical model and (b) the water diffusion coefficient as a function of viscosity for different pressures.

(4) F. H. Stillinger, Science **209**, 451 (1980).

(5) C. Tanford, *The hydrophobic effect: formation of micelles and biological membranes*, 2nd ed. (Wiley, New York, 1980).

(6) G. Robinson, S.–B. Zhu, S.Singh and M. Evans, *Water in Biology, Chemistry and Physics: Experimental Overviews and Computational Methodologies* (World Scientific, Singapore, 1996).

(7) L. R. Pratt, Ann. Rev. Phys. Chem. **53** 409 (2002).

(8) B. Widom, P. Bhimalapuram and K. Koga, Phys. Chem. Chem. Phys. **5**, 3085 (2003).

(9) J. H. Griffith and H. A. Scheraga, J. Molec. Struc. (Theochem), **682**, 97 (2004).

(10) D. M. Huang and D. Chandler, J. Phys. Chem. B **106**, 2047, (2002).

(11) M. Predota, A. Ben-Naim and I. Nezbeda, J. Chem. Phys. **118**, 6446 (2003).

(12) I. Nezbeda, J. Mol. Liquids **73/74**, 317 (1997).

(13) D. E. Smith and A. D. J. Haymet *in Reviews in Computational Chemistry* ed. K. B. Lipkowitz, R. Larter, and T. R. Cundari (Wiley-VCH,New York, 2003).

(14) K.A. Dill, T.M. Truskett, V. Vlachy, and B. Hribar-Lee, Ann. Rev. Biophys. Biomolec. Struc. **34**, 173 (2005).

(15) S.-H. Chen, F. Mallamace, C.-Y. Mou, M. Broccio, C. Corsaro, A. Faraone, L. Liu, Proc. Natl. Acad. Sci. U.S.A. **103**, 12974–12978 (2006).

(16) P. Kumar, Proc. Natl. Acad. Sci. U.S.A. **103**, 12955–12956 (2006).

(17) P. Kumar, S. V. Buldyrev, S. R. Becker, P. H. Poole, F. W. Starr, H. E. Stanley, Proc. Natl. Acad. Sci. U.S.A. **103**, 9575–9579 (2006).

(18) L. Xu, P. Kumar, S. V. Buldyrev, S.-H. Chen, P. Poole, F. Sciortino, H. E. Stanley, Proc. Natl. Acad. Sci. U.S.A. **102**, 16558–16562 (2005).

(19) P. Kumar, G. Franzese, H. E. Stanley, Phys. Rev. Lett. **100**, 105701 (2008).

(20) C. A. Angell, J. Non-Cryst. Solids **13**, 131–133 (1991).

(21) C. A. Angell, Science **67**, 1924 (1995).

(22) T. Urbic and K. A. Dill, J. Chem. Phys. **132**, 224507 (2010).

(23) T. Urbic, Phys. Rev. E. **85**, 061503 (2012).

(24) T. Urbic, Phys. Rev. E. **94**, 042126 (2016).

(25) A. Ben-Naim, J. Chem. Phys. **54**, 3682 (1971).

(26) A. Ben-Naim, Mol. Phys. **24**, 705 (1972).

(27) A. Ben-Naim, *Water and Aqueous Solutions* (Plenum Press, New York, 1974).

(28) A. Ben-Naim, *Molecular Theory of Water and Aqueous Solutions, 1st ed.* (World Scientific, Singapore, 2009).

(29) T. M. Truskett and K. A. Dill, J. Chem. Phys., **117**, 5101 (2002).

(30) T. M. Truskett and K. A. Dill, J. Phys. Chem. B, **106**, 11829 (2002).

(31) E. A. Jagla, J. Chem. Phys. **111**, 8980 (1999).

(32) K. A. T. Silverstein, A. D. J. Haymet and K. A. Dill, J. Am. Chem. Soc. **120**, 3166 (1998).

(33) F. Mallamace, C. Corsaro, D. Mallamace, C. Vasic, H. E. Stanley, Faraday Discuss. **167**, 95–108 (2013).

(34) K. R. Harris and P. J. Newitt, J. Chem. Eng. Data **42**, 346–348 (1997).

(35) K. Krynicki, C. D. Green and D. W. Sawyer, Faraday Discuss. Chem. Soc., **66**, 199-208 (1978).

(36) T. Urbic, K. A. Dill, Phys. Rev. E, **98**, 032116 (2018).

(37) K. E. Bet and J. B. Cappi, Nature **207**, 620–621 (1965).

(38) T. DeFries, and J. Jonas, J. Chem. Phys. **66** 896–901 (1977).

(39) X. Y. Liu, M. G. He and Y. Zhang, J Supercrit Fluids **63**, 150-154 (2012).

(40) J. V. Sengers, J. T. R. Watson, R. S. Basu, B. Kamgar-Parsi and R. C. Hendricks, J. Phys. Chem. Data **13**, 893 (1984).

(41) E. H. Abramson, J. Chem. Phys. **115**, 10461 (2001).
